# Potential immune of recombinant serine protease of *Corynebacterium pseudotuberculosis*

**DOI:** 10.1186/1753-6561-8-S4-P143

**Published:** 2014-10-01

**Authors:** Daniela Droppa Almeida, Wanessa Lordelo Vivas, Judson Wallace Rodrigues da Silva, Caroline de Santana Ferreira, Katharina Kelly Oliveira, Ioná Brito, Fernando Mendonça Diz, Sibele Borsuk, Isabel Bezerra Lima-Verde, Vasco Azevedo, Roberto Meyer, Francine Ferreira Padilha

**Affiliations:** 1 Universidade Tiradentes - Unit, Aracaju, Brazil; 2 Universidade Estadual do Ceará, Fortaleza, Brazil; 3 Universidade Federal de Pelotas - UFPel, Pelotas, Brazil; 4 Universidade Federal de Minas Gerais - UFMG, Belo Horizonte, Brazil; 5 Universidade Federal da Bahia - UFBA, Salvador, Brazil

## Background

Caseous lymphadenitis (CLA) has high relevance. It is a chronic disease that affects sheep and goats. The causative agent is *Corynebacterium pseudotuberculosis*, facultative intracellular bacteria. The prevalence of caseous lymphadenitis is high in many regions of the world, including South America. Brazil has 78% of seroprevalence in goats [1,2]. Therefore, prophylaxis becomes the best strategy as an effective vaccine able to eradicate disease, but also to formulate a serologic test able to detect visceral cases. One of the first steps for developing of a serological test and an effective vaccine is the choice of a target, which must present appropriate antigenic characteristics. The CP40 protein (corynebacterial protease 40) have been studied in several studies. This protease is considered one of the virulence factors of *C. pseudotuberculosis*. Encoded by the gene CP40 and characterized as a serine protease, proteins responsible for several functions, such as providing the activation of pro-inflammatory citocines which will help in the activation of the immune response. However the aim of this study was to evaluate the potential immune serine protease recombinant in BALB/c mice.

## Methods

To this end, CP40 protein was expressed in a heterologous genes in a prokaryotic system and purified by nickel affinity chromatography. The antigenicity of rCP40 was determined through positive and negative sera from animals for LC by Western Blotting. To determine immunogenicity, BALB / c mice were inoculated with a solution of rCP40, saponin and Freund's adjuvants in three doses on days 0, 15 and 30. The serum samples of these animals were collected to check the production of specific antibodies rCP40. Statistical analysis was performed using GraphPad Prism version 6.0 for Windows (GraphPad Software, San Diego, CA).

## Results and conclusions

Positive sera reacted with the recombinant protein, demonstrating that rCP40 maintained the antigenic properties, which is important because it demonstrates that antibodies generated against the native protein present in the bactéria *C. pseutuberculosis *were able to recognize the recombinant protein. The results of the ELISA showed that the development of humoral immune response was generated by the experimental groups were inoculated with rCP40 associated with both adjuvants. The activation of helper T cells express ligands. Cytokines induce isotype switching of immunoglobulins [3] which are fixed more firmly to the antigens and plays diverse functions, forming the most effective mediators of humoral response by involving the cellular immune system, including phagocytosis and complement proteins. In mice the role of CD4 + T-cells helper can provide production class antibody IgG2a and IgG2B that are capable of promoting directly or indirectly a response from Th1 and lead to opsonization and toxicity mediated by cells [4].
